# Gly-tRF enhances LCSC-like properties and promotes HCC cells migration by targeting NDFIP2

**DOI:** 10.1186/s12935-021-02102-8

**Published:** 2021-09-18

**Authors:** Yongqiang Zhou, Jinjing Hu, Lu Liu, Mengchao Yan, Qiyu Zhang, Xiaojing Song, Yan Lin, Dan Zhu, Yongjian Wei, Zongli Fu, Liming Hu, Yue Chen, Xun Li

**Affiliations:** 1 The First Clinical Medical College of Lanzhou University, Lanzhou University, 222 Tianshui South Road, Lanzhou, 730000 China; 2 Department of General Surgery, The First Hospital of Lanzhou University, Lanzhou, 730000 China; 3 Gansu Province Key Laboratory of Biotherapy and Regenerative Medicine, Lanzhou, 730000 China; 4 School of Life Science of Lanzhou University, Lanzhou University, Lanzhou, 730000 China

**Keywords:** Hepatocellular carcinoma, Liver cancer stem cells, tRNA-derived fragments, NDFIP2, EMT, AKT

## Abstract

**Background:**

Accumulating evidence demonstrates that tRFs (tRNA-derived small RNA fragments) and tiRNAs (tRNA-derived stress-induced RNA), an emerging category of regulatory RNA molecules derived from transfer RNAs (tRNAs), are dysregulated in in various human cancer types and play crucial roles. However, their roles and mechanisms in hepatocellular carcinoma (HCC) and liver cancer stem cells (LCSCs) are still unknown.

**Methods:**

The expression of glycine tRNA-derived fragment (Gly-tRF) was measured by qRT-PCR. Flow cytometric analysis and sphere formation assays were used to determine the properties of LCSCs. Transwell assays and scratch wound assays were performed to detect HCC cell migration. Western blotting was conducted to evaluate the abundance change of Epithelial-mesenchymal transition (EMT)-related proteins. Dual luciferase reporter assays and signalling pathway analysis were performed to explore the underlying mechanism of Gly-tRF functions.

**Results:**

Gly-tRF was highly expressed in HCC cell lines and tumour tissues. Gly-tRF mimic increased the LCSC subpopulation proportion and LCSC-like cell properties. Gly-tRF mimic promoted HCC cell migration and EMT. Loss of Gly-tRF inhibited HCC cell migration and EMT. Mechanistically, Gly-tRF decreased the level of NDFIP2 mRNA by binding to the NDFIP2 mRNA 3′ UTR. Importantly, overexpression of NDFIP2 weakened the promotive effects of Gly-tRF on LCSC-like cell sphere formation and HCC cell migration. Signalling pathway analysis showed that Gly-tRF increased the abundance of phosphorylated AKT.

**Conclusions:**

Gly-tRF enhances LCSC-like cell properties and promotes EMT by targeting NDFIP2 and activating the AKT signalling pathway. Gly-tRF plays tumor-promoting role in HCC and may lead to a potential therapeutic target for HCC.

**Supplementary Information:**

The online version contains supplementary material available at 10.1186/s12935-021-02102-8.

## Background

Hepatocellular carcinoma (HCC) is one of the most common malignant tumours, causing a substantial global health burden [1]. Reasonable methods of prevention, monitoring, early detection, diagnosis and treatment have been developed [2], however, the survival of HCC patients after radical resection is poor [3]. Investigation of the underlying mechanisms of HCC invasiveness and metastasis is of great significance for finding new therapeutic targets that can improve the prognosis of HCC.

Newly discovered types of noncoding RNAs (ncRNAs) derived from pre-transfer RNA (tRNA) or mature tRNA by precise site-specific cleavage are tRFs (tRNA-derived small RNA fragments) and tiRNAs (tRNA-derived stress-induced RNA) [4]. Abnormal expression of tRFs and tiRNAs has been observed in many diseases, including tumours, neurodegenerative diseases, and metabolic and infectious diseases [5, 6]. tRFs and tiRNAs have been detected in a variety of body fluids and tissues [7], and their expression are highly abundant [8, 9], heavily modified and not easily degraded [10]; thus, they are more stable than other ncRNAs and increasingly becoming a popular topic in oncology research [11]. Accumulating evidence shows that tRFs and tiRNAs play crucial roles in human cancers, including breast cancer [12–15], prostate cancer [16, 17], and colorectal cancer [18, 19], by participating in multiple biological functions, including gene expression and silencing, translation regulation and epigenetic regulation [20].

A recent study showed that glycine tRNA-derived fragment (Gly-tRF) expression is upregulated in ethanol-fed mice and promotes alcoholic fatty liver disease (AFLD) [21]. AFLD is one of the early forms of liver injury. Some patients with simple steatosis can develop more severe forms of liver injury, including steatohepatitis, cirrhosis, and eventually HCC [22]. Here we aimed to explore the impact of Gly-tRF on the biological process of HCC and the roles of Gly-tRF in LCSC.

In the present study, Gly-tRF was found to be upregulated in HCC tissues and cell lines, and increased expression of Gly-tRF triggers EMT and the acquisition of LCSC-like properties.

Furthermore, target genes prediction and Dual luciferase reporter assays indicated that NDFIP2 was a direct target of Gly-tRF. Subsequently, we observed that overexpression of NDFIP2 weakened the promotive effects of Gly-tRF on EMT and LCSC-like cell sphere formation ability. Finally, bioinformatics analysis indicated that Gly-tRF functions by activating the AKT signalling pathway (A flowchart of the article is shown in Additional file 1: Figure S1). Therefore, this study illustrates that Gly-tRF plays tumor-promoting role in HCC and may lead to a potential therapeutic target for HCC.

## Materials and methods

### Specimen collection, tissue microarray and immunohistochemical staining

Fifteen samples of histologically confirmed tumours and matched adjacent non-tumour tissues obtained from HCC patients who underwent radical hepatectomy at Lanzhou University First Hospital. The study was approved by the hospital ethics committee, and according to the institutional review committee's procedures, all patients signed an informed consent form before the study. A tissue microarray containing 90 tumour tissues and matched adjacent non-tumour tissues was purchased from Shanghai Outdo Biotech Co., Ltd (Shanghai, China). All patients provided written informed consent and were followed up for 5–6 years with clear prognostic information. Immunohistochemical staining was performed as previously described [23]. For immunohistochemical images, two experienced pathologists independently performed immunohistochemical staining scores according to the staining intensity (0: no staining; 1: weak staining; 2: moderate staining; 3: strong staining) and the percentage of positive cells (0: 0%; 1: < 25%; 2: 26–50%; 3: > 50%). The staining intensity score and the staining percentage score were summed to calculate the final immunohistochemical score. We defined an immunohistochemical of score 0 to 4 as low expression and a score of 5 to 6 as high expression.

### Cell culture

The human liver cancer cell lines HepG2, Huh7 and HCCLM3 were purchased from the China Center for Type Culture Collection (CCTCC, Wuhan, China) and were identified by short tandem repeat (STR). L02 hepatocytes were gift from Zhongshan Hospital of Fudan University (Shanghai, China). The embryonic kidney cell line HEK-293 T was a gift from Shanghai GeneChem Co., Ltd. (Shanghai, China). All cells were grown in Dulbecco’s modified Eagle’s medium (DMEM; pH = 7.2, Gibco Company, Grand Island, NY, USA) containing 10% (v/v) foetal bovine serum (FBS, HyClone, Logan, UT, USA). All cells were cultured in a humidified incubator (Thermo Fisher Scientific, Waltham, MA, USA) at 37 °C and 5% CO2. All cells were tested for mycoplasma contamination.

### RNA isolation

Total RNA was harvested from cells and tissues using RNAiso Plus (Takara Holdings Inc., Kyoto, Japan), and the protocol recommended by the manufacturer protocol was followed for isolation of total RNA. NanoDrop 2000 (Thermo Fisher Scientific) was used to measure the quality and quantity of the isolated RNA.

### 3′ and 5′ Adaptor ligation, first-strand complementary DNA (cDNA) synthesis and real-time PCR

Heavy modifications contained in tRNAs, such as 3′-aminoacyl, 3′-cP, m1A, m1G, and m3C modifications will severely interfere with reverse transcription. Therefore, conventional PCR methods may not be able to reflect the true expression characteristics of tRNA-derived fragments [24]. In this work, an rtStar™ tRF&tiRNA Pretreatment Kit (Arraystar Inc., Rockville, MD, USA. Cat #AS-FS-005) was used to remove various modifications from Gly-tRF before 3′ and 5′ adaptor and cDNA synthesis. cDNA was synthesized using APExBIO First-strand cDNA Synsthesis Supermix (APExBIO Inc., Houston, TX, USA; Cat #K1073). All steps, such as 3′-terminal deacylation, 3′-cP removal and 5′-P addition, demethylation and reverse transcription, were carried out in accordance with the manufacturer's instructions. All reactions were performed in an Mx3000P QPCR system (Agilent Technologies Inc., Santa Clara, CA, USA) using TB Green Premix Ex Taq II (Takara Holdings Inc., Kyoto, Japan) for real-time PCR according to the manufacturer's instructions. The primers are listed in Table 1. U6 or GAPDH was used as the normalized endogenous control for expression. The relative expression levels of Gly-tRF were analysed by the 2^−ΔΔCt^ method.

**Table 1:** Sequences information in this study

Genes	Sequences
Gly-tRF	GCAUUGGUGGUUCAGUGGUAGAAUUCUCGC
	Forward: CATTGGTGGTTCAGTGGTAGAAT Reverse: AGTGCAGGGTCCGAGGTATT
Gly-tRF NC-inhibitor	TTCTCCGAACGTGTCACGT
Gly-tRF inhibitor	GCGAGAATTCTACCACTGAACCACCAATGC
Gly-tRF NC-mimic	CON238
Gly-tRF mimic	GCCTTGTTAAGTGCTCGCTTCGGCAGCACATATACTATGTTTGAATGAGGCTTCAGTACTTTACAGAATCGTTGCCTGCACATCTTGGAAACACTTGCTGGGATTACTTCTTCAGGTTAACCCAACAGAAGGCTCGAGAAGGTATATTGCTGTTGACAGTGAGCGACGAGAATTCTACCACTGAACCACCAATGCTAGTGAAGCCACAGATGTAGCATTGGTGGTTCAGTGGTAGAATTCTCGCTGCCTACTGCCTCGCAATTCAAGGGGCTACTTTAGGAGCAATTATCTTGTTTACTAAAACTGAATACCTTGCTATCTCTTTGATACATTTTTACAAAGCTGAATTAAAATGGTATAAATTAAATCACTTTTTTCAATTGGAAGACTAATGCGTTTAAACACGCGGCG
NDFIP2	Forward: TCAAACCCAGCACCGCAGATTG Reverse: CGCAGATAGCACCATACCTTCCAG
ABHD17B	Forward: GCTGCTTGGCTTGCTCTTAGGAC Reverse: TTCAACCCAGAGAGGCTCCACAG
KCNK10	Forward: ATGAAGTGGAAGACGGTGGTTGC Reverse: AGTGGCTGCTGTTGTTGGAAGAG
RNF103	Forward: TCATGGGTAAGGGCAGACTGGATG Reverse: AAAGAAGCAATCGGGTGGAAGAGG
CXXC4	Forward: TCCTCCTCCGCCTCCTCCTC Reverse: TGGCAATTTGAAACGCACTGTCTG
OXTR	Forward: GGTGGTGGCAGTGTTTCAGGTG Reverse: CAGGCAGCGAGCACGATGAC
WDR44	Forward: CAGTGGAAGTCAAAGGAGGTGGTG Reverse: GCCATGCTTGCGGTTAGGAGAG
OSER1	Forward: AGCACCAGTCAGAACAGCAACAG Reverse: TTGGGTAGCGTCAGAGGAGTCTTC
GAPDH	Forward: CCCACTAACATCAAATGGGG Reverse: CCTTCCACAATGCCAAAGTT
U6	Forward: CGCTTCGGCAGCACATATAC Reverse: GAACGCTTCACGAATTTGCGT

### Cell transfection

HCCLM3 and Huh7 cells were seeded in 6-well plates (Corning Life Sciences, USA) at a density of 4 × 10^5^ cells per well. When the cells were 40–50% confluent, Gly-tRF negative control, Gly-tRF inhibitor, and Gly-tRF mimic lentiviruses were used to transduce cells according to the manufacturer's instructions (Shanghai GeneChem Co., Ltd, Shanghai, China). The sequences of all lentiviral products are shown in Table 1. After 72 h of transduction, cells were cultured in complete medium containing 2 µg/µL puromycin for 72 h. Cells stably transduced with Gly-tRF were used for subsequent experiments.

### Proportional staining analysis of representative LCSC markers

HCC cells were prepared as single-cell suspensions for staining. All antibodies used for staining were purchased from Miltenyi Biotec (Bergisch Gladbach, Germany), and included a phycoerythrin (PE)-conjugated anti-CD133 antibody (Cat #130-110-962), a PE-Vio770-conjugated anti-CD13 antibody (Cat #130-120-727), an allophycocyanin (APC)-conjugated anti-EpCAM antibody (Cat #130-111-000), an APC-Vio770-conjugated anti-CD44 antibody (Cat #130-113-339) and anti-REA control antibody (Cat # 130-113-438, 130-113-440, 130-113-434, 130-113-445). The percentages of CD133^+^, CD13^+^, EpCAM^+^, and CD44^+^ within the HCC cell population were determined according to the manufacturer’s instructions. In brief, 1 × 10^6^ cells were centrifuged and resuspended in 98 µL of buffer, 2 µL antibody was added, and the cells were incubated for 10 min in the dark at 4 °C. The cells were washed with 1 mL of buffer, centrifuged at 300 g for 10 min, and resuspended in 400 µL of buffer for detection. Data were acquired with a BD LSRFortessa. All samples were analysed in triplicate.

### Sphere formation assays

Cells (2000 cells per well) were planted in a 6-well ultra-low adhesion plate (Corning, USA). After 8 days of incubation, spheres were counted and photographed (15 random fields/well) under a stereomicroscope (Olympus, Tokyo, Japan). The diameter of the spheres was measured with Image-Pro Plus 6.0 software (Media Cybernetics Inc., Rockville, MD, USA), and colonies with a diameter greater than 20 µm were considered positive for sphere formation.

### Transwell assays

Cell migration experiments were performed in a 24-well Transwell plate (8.0 µm pore size, Corning Life Sciences, Costar, USA). Stably transduced cells were starved for 6 h in serum-free medium, trypsinized and adjusted to 2 × 10^5^ cells/mL after counting. Then, 600 µL of complete medium containing 30% (v/v) serum was added to the lower chamber, 200 µL of the cell suspension was added to the upper chamber, and the cells were cultured for 48 h. The cells in the upper chamber were removed, and the cells remaining on the membrane were fixed with 4% paraformaldehyde (Solarbio, Beijing, China). After staining with 0.5% crystal violet (Solarbio, Beijing, China), the cells were observed under a microscope and imaged. All experiments were repeated three times.

### Wound healing assay

Stably transduced cells were trypsinized and seeded in a 6-well plate. When the cells were 90% confluent, a 200 µL sterile pipette tip was used to uniformly make vertical scratches in the wells of the 6-well plate. Cells were removed by washing 3 times with PBS and multiple random fields were selected to observe cell migration at 0 h, 24 h, and 48 h. The area and width of the scratches were quantified with Image-Pro Plus 6.0.

### Protein extraction and western blot analysis

Western blotting was performed as previously described [25]. The primary antibodies used for Western blotting were as follows: anti-NDFIP2(1:1000, Bioss Antibodies Inc., Beijing, China; Cat # bs-19059R), anti-pan AKT (1:1000, Abcam, Cambridge, UK; Cat # ab8805), anti- phospho-AKT1 (1:1000, Abcam, Cat # ab66138), anti-N-cadherin (1:1000, Abcam, Cat # ab18203), anti-E-cadherin (1:10,000, Abcam, Cat # ab40772). An anti-β-actin antibody (1:2000, Sigma, USA) was used as an internal control to ensure equal amounts of protein loading.

### Immunofluorescence staining

Stably transduced cells were grown overnight on glass coverslips. The cells were fixed with 4% paraformaldehyde for 10 min at room temperature. The cells were washed 3 times with -cold PBS. The cells were then incubated with PBS (containing 0.3% Triton X-100) for 10 min. The cells were washed 3 times with PBS for 5 min each. The cells were blocked 3% BSA for 30 min at room temperature. The cells were then incubated with an anti-NDFIP2 antibody (1:200, Bioss Antibodies Inc., Beijing, China; Cat # bs-19059R) overnight at 4 °C. The cells were washed 3 times with PBS-T, and were then incubated with Cy3-conjugated goat anti-rabbit (1:400, Servicebio, Wuhan, China; Cat # GB21303) at room temperature in the dark for 60 min. The cells were then incubated with 4′,6-diamidino-2-phenylindole (DAPI, Servicebio) for 5 min. After washing with PBS, images were acquired using a fluorescence microscope (Nikon Eclipse C1; Nikon Corporation). Fluorescence quantitative analysis was performed using Image-Pro Plus 6.0.

### Plasmid construction, plasmid transfection and luciferase assay

pcDNA3.1 was used as the vector to construct the NDFIP2 overexpression plasmid (pcDNA3.1 + NDFIP2 OE), and pGL6 (Beyotime, Shanghai, China) was used as the vector to construct the NDFIP2 3' UTR wild-type (NDFIP2 wt) and NDFIP2 3' UTR mutant (NDFIP2 mut) luciferase reporter plasmids. All constructed plasmids were verified by sequencing (TSINGKE, Beijing, China). The Renilla luciferase reporter plasmid pRL-TK and the pGL6 promoter empty vector were co-transfected with NDFIP2 wt or NDFIP2 mut into the HEK-293T cells in each well using Exfect Transfection Reagent (Vazyme Biotechnology Co., Ltd, Nanjing, China) following the manufacturer's instructions. In brief, 50 ng of pRL-TK and 400 ng of NDFIP2 wt, NDFIP2 mut or pGL6 were added to Opti-MEM, mixed with 1 µL liposomes and incubated for 10 min at room temperature. Forty-eight hours after transfection, the cells were lysed, and a Dual-Luciferase® reporter analysis system (Promega, Madison, WI, USA) was used to perform dual-luciferase reporter assays. A GLOMAX 20/20 luminometer (Promega, Madison, WI, USA) was used to detect luciferase activity. All samples were analysed in triplicate. The same method was used to transfect HCCLM3 cells with pcDNA3.1 + NDFIP2 OE and pcDNA3.1 empty vector (pcDNA3.1 + vector). In brief, 3 µg of pcDNA3.1 + NDFIP2 OE or pcDNA3.1 + vector and 9 µL of liposomes were added to the cells. After 48 h of culture, follow-up experiments were performed.

### Bioinformatic analysis

Gene expression profiles and clinical data were downloaded from the TCGA XENA database (https://xena.ucsc.edu/) to identify differentially expressed genes (DEGs) between HCC tissues and matched non-tumour tissues. Gene Ontology (GO) enrichment analysis was performed with the for DEGs.

### Statistical analysis

GraphPad Prism 8 (La Jolla, CA, USA) was used for all statistical analysis and plotting. P < 0.05 was considered statistically significant. Student’s *t*-test or one-way ANOVA was used for intergroup comparisons of quantitative data.

## Results

### Gly-tRF expression is elevated in HCC

**Fig. 1:**
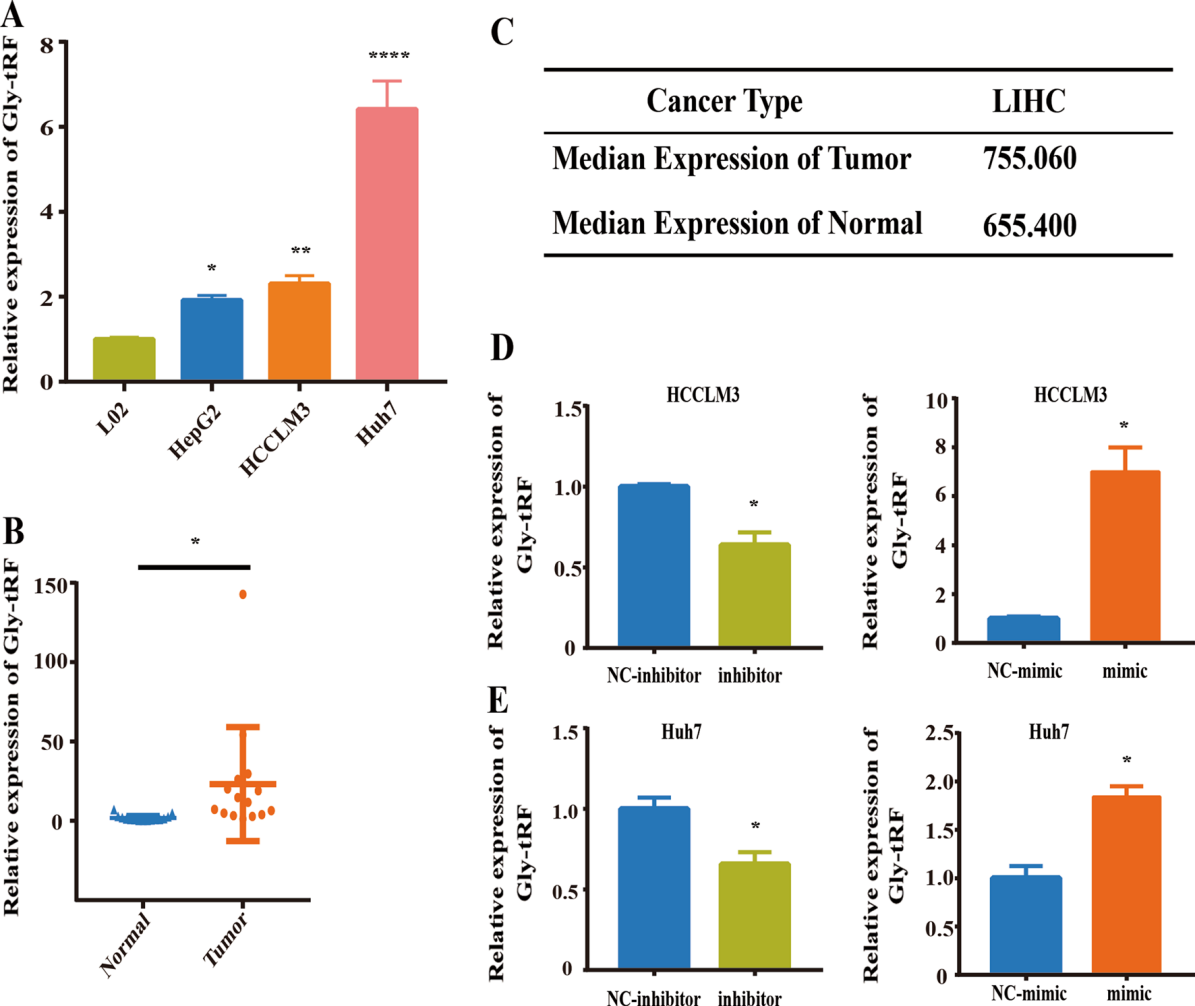
Gly-tRF expression is elevated in HCC. **A** Quantitative real-time PCR (qRT-PCR) shows that Gly-tRF is highly expressed in HCC cell lines (HCCLM3, Huh7, HepG2), compared to L02 hepatocytes. **B** Scatter plots shows that in HCC specimens, the expression of Gly-tRF in tumor tissues is elevated compared with adjacent non-tumour ones (n = 15). **C** Based on the online database OncotRF, the median expression level of Gly-tRF in tumour tissues of HCC patients is higher than that in normal tissues. LIHC: Liver hepatocellular carcinoma. **D**, **E** Confirming of Gly-tRF enhancement or reduction transfected with Gly-tRF mimic or Gly-tRF inhibitor by qRT-PCR in HCCLM3 and Huh7 cells. Data are shown as mean ± SD. *P < 0.05, **P < 0.01, ****P < 0.0001

The differential expression of Gly-tRF in L02 hepatocytes and HCC cell lines (HCCLM3, Huh7, HepG2) by were determined qRT-PCR. The expression of Gly-tRF in HCC cell lines was higher than that in L02 cells (Fig. 1A). In the 15 HCC specimens, the expression of Gly-tRF in tumour tissues was elevated compared with that in adjacent non-tumour tissues (Fig. 1B). Based on the online database OncotRF (http://bioinformatics.zju.edu.cn/) [26], the median expression level of Gly-tRF in tumour tissues of HCC patients was higher than that in normal tissues (755.06RPM vs 655.40RPM) (Fig. 1C). The abnormal expression of Gly-tRF in HCC suggested that it might be involved in HCC progression. To evaluate the effect of Gly-tRF on HCC cell functions, Gly-tRF negative control, Gly-tRF inhibitor, and Gly-tRF mimic lentiviruses were transduced into HCCLM3 and Huh7 cells. We used qRT-PCR to confirm the Gly-tRF inhibitor blocked the expression of Gly-tRF in HCCLM3 and Huh7 cells (Fig. 1D, Fig. 1E). In addition, whether Gly-tRF mimic transduction boosts Gly-tRF expression was assessed (Fig. 1D, Fig. 1E).

### Gly-tRF is involved in the maintenance of LCSC-like properties

**Fig. 2:**
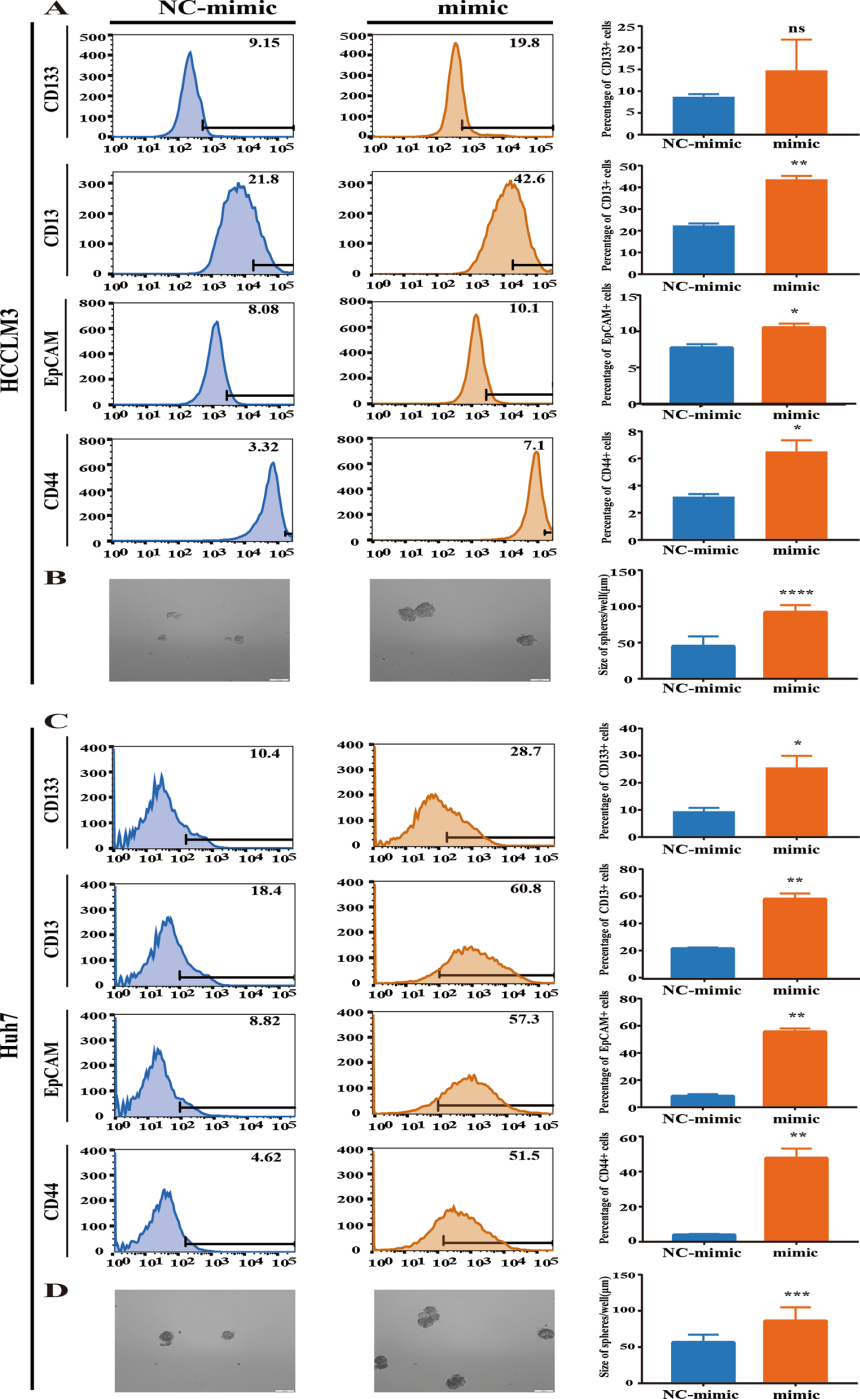
Gly-tRF is involved in the maintenance of LCSC-like properties. **A**, **C** Flow cytometric analysis to detect the percentage of representative LCSC surface markers (CD133, CD13, EpCAM, CD44) in HCCLM3 and Huh7 cells stably transfected with Gly-tRF NC-mimic and Gly-tRF mimic. The statistical graph shows result of three independent experiments. **B**, **D** Sphere formation assay to reflect the sphere formation ability of those cells with LCSC-like properties in HCCLM3 and Huh7 transfected with Gly-tRF NC-mimic and Gly-tRF mimic. Scale bar = 100 μm. The statistical graph shows the diameter of spheres in three independent experiments. Data are shown as mean ± SD. ns means no significance, *P < 0.05, **P < 0.01, ***P < 0.001, ****P < 0.0001

The existence of liver cancer stem cells (LCSCs) is generally considered to be the primary cause of HCC metastasis, malignant growth and treatment failure [27]. We investigated whether Gly-tRF has any effects on LCSCs. LCSC surface markers (CD133/CD13/EpCAM/CD44) is often considered to represent the LCSC population [28]. We compared the percentages of CD133^+^, CD13^+^, EpCAM^+^ and CD44^+^ in HCCLM3 and Huh7 cells transduced with Gly-tRF mimic using flow cytometry. Interestingly, our results showed that HCCLM3 cells stably transduced with the Gly-tRF mimic had higher percentages of CD13^+^, EpCAM^+^ and CD44^+^ (Fig. 2A). However, the percentage of CD133^+^ cells showed no significant difference attributable to discrete detection data (Fig. 2A). Similarly, Gly-tRF elevated the percentages of CD133^+^, CD13^+^, EpCAM^+^ and CD44^+^ in Huh7 cells (Fig. 2C). This result suggests that Gly-tRF expression may be involved in the regulation of LCSC.

Sphere formation assay is a widely used model for studying CSCs [29]. Next, the effect of Gly-tRF on the sphere formation ability was further investigated. Our study reveals that cells transduced with the Gly-tRF mimic had a higher LCSC sphere formation ability (Fig. 2B, D). These findings indicate that in human HCC, Gly-tRF plays a role in the maintenance of LCSC-like properties.

### Gly-tRF intensifies HCC cell migration and EMT

Convincing evidence shows that the existence of CSCs is responsible for EMT in cancer cells [30]. In addition, a large amount of credible evidence shows the driving role of CSCs in tumour migration [31]. Therefore, we continue to evaluate the effect of Gly-tRF on the migration of HCC cells.

**Fig. 3:**
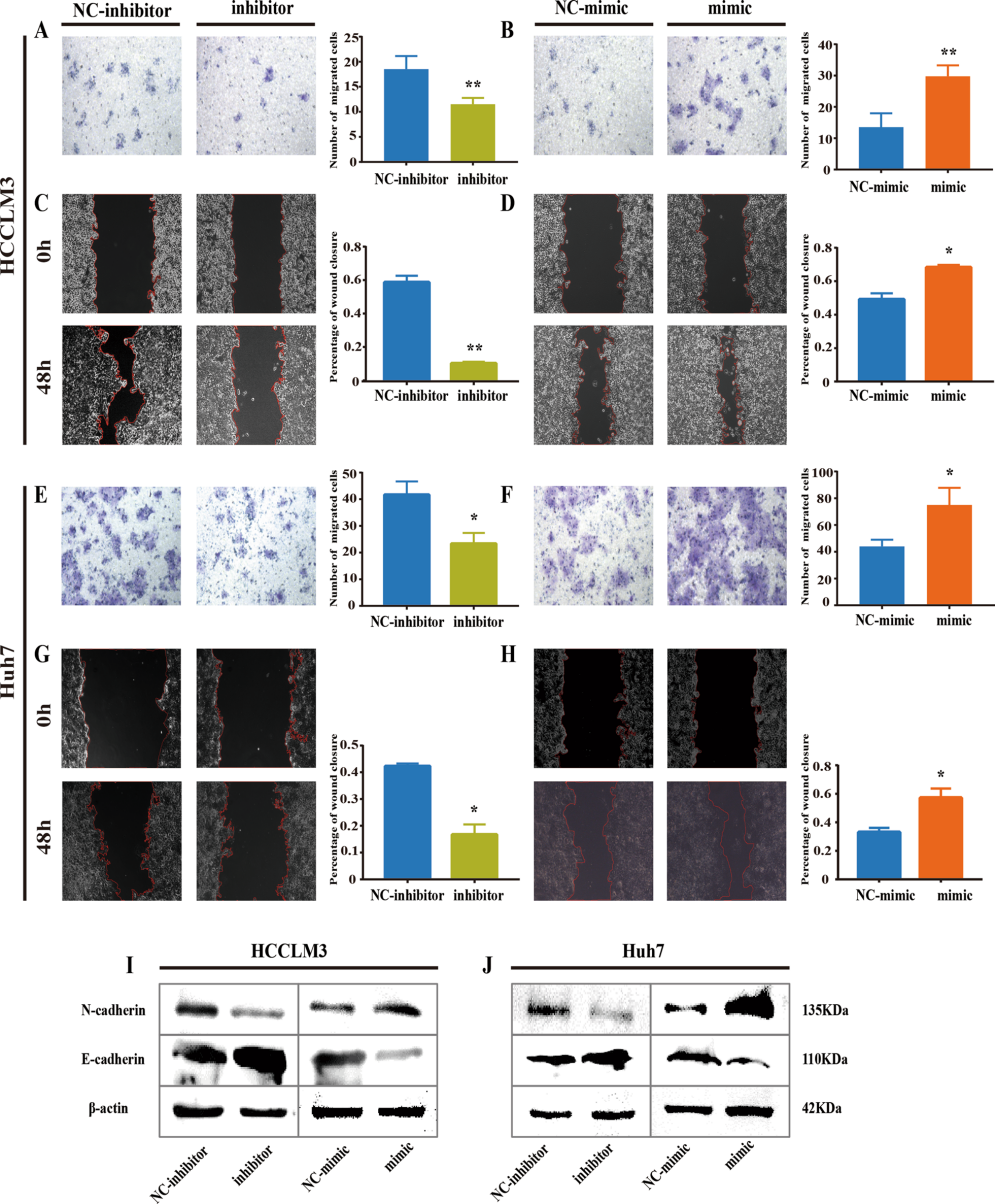
Gly-tRF intensifies migration and EMT of HCC cells. **A**, **B**, **E**, **F** Transwell assay performed to evaluate cell migration in HCCLM3 and Huh7 cells after Gly-tRF mimic or Gly-tRF inhibitor transduction. The statistical graph shows the average number of migrated cells in 5 random fields. Magnification, 200 × . **C**, **D**, **G**, **H** Wound healing assay was used to confirm the effect of Gly-tRF on HCC cells migration. The percentage of wound closure represents the effect of Gly-tRF on the migration ability of cancer cells. Magnification, 100 × . Data are shown as mean ± SD of three independent experiments. ns means no significance, *P < 0.05, **P < 0.01. **I**, **J** Western blotting was performed to detect the abundance of core markers (N-cadherin, E-cadherin) in EMT

Through Transwell assays, we observed that the number of migrated cells increased after Gly-tRF mimic transduction and decreased after Gly-tRF inhibitor transduction (Fig. 3A, B, E, F) in HCCLM3 and Huh7 cells.

Wound healing assay was used to confirm the effect of Gly-tRF on HCC cell migration. Our results showed that Gly-tRF mimic transduction accelerated wound healing, while Gly-tRF inhibitor transduction slowed wound healing at 48 h, (Fig. 3C, D, G, H). These data suggest that Gly-tRF inhibits migration in HCC.

EMT allows cancer cells to infiltrate into the surrounding tissues and metastasizes [32], cancer cell migration is often accompanied by EMT. Next, we used Western blotting to detect the abundance of key markers of EMT. The results showed that in the cells transduced with the Gly-tRF inhibitor, the abundance of N-cadherin decreased, while the abundance of E-cadherin increased. It was also observed that Gly-tRF mimic transduction correspondingly reversed this change (Fig. 3I, J). These results demonstrate that Gly-tRF as a cancer-promoter, plays critical roles in HCC by promoting cell migration and EMT.

### Gly-tRF inhibits NDFIP2 3′ UTR-driven luciferase reporter activity

Studies have shown that tRNA-derived fragments inhibit the biological functions by binding to the mRNA 3′ UTR of the target gene and exerting effect similar to that of miRNAs [33, 34]. The online database miRDB allows the use of user-provided tRNA-derived fragment sequences for custom target prediction [35]. To investigate the regulatory effect of Gly-tRF on downstream genes, we used miRDB to predict several Gly-tRF target genes (Table. 2).

**Table 2:** The top 8 Gly-tRF target genes predicted by miRDB

Gene	Gene description	Predicted target score	3´UTR length	Seed position
ABHD17B	Abhydrolase domain containing 17B	96	129	82
NDFIP2	Nedd4 family interacting protein 2	96	3564	32
KCNK10	Potassium two pore domain channel Subfamily K member 10	95	5427	676
RNF103	Ring finger protein 103	95	1564	896
CXXC4 OXTR	CXXC finger protein 4 Oxytocin receptor	95 95	4106 2569	2120, 2783 1560, 2131, 2537
WDR44	WD repeat domain 44	94	974	860
OSER1	Oxidative stress responsive serine rich 1	94	1113	29

**Fig. 4:**
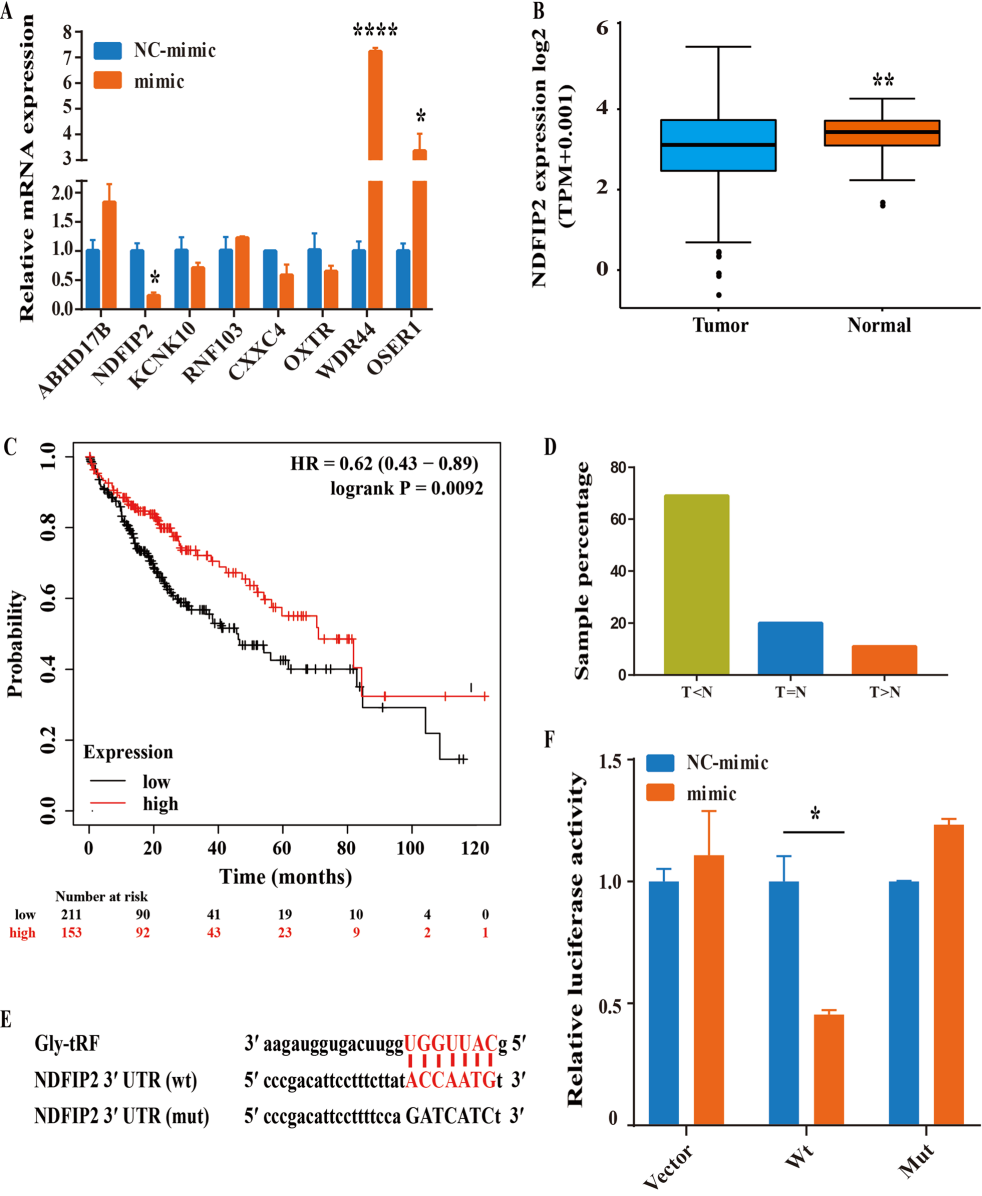
Gly-tRF inhibits NDFIP2 3′ UTR luciferase reporter activity. **A** qRT-PCR was used to detect the mRNA expression of target genes predicted by miRDB in HCCLM3 cells transfected with Gly-tRF mimic. **B** Expression of NDFIP2 gene in 371 HCC tissues and 50 normal liver tissues from the TCGA database. **C** The expression of NDFIP2 is related to the prognosis of HCC Kaplan–Meier Plotter database. **D** The expression of NDFIP2 in HCC was verified by immunohistochemistry. **E** The sequence of Gly-tRF and the binding site of NDFIP2 3′UTR are shown. The sequence of the NDFIP2 mutant plasmid is also listed. **F** Luciferase activity of cells transfected with pGL6 empty vector(vector), NDFIP2 3′ UTR wild-type(wt) and mutant plasmids(mut) co-transfected with Gly-tRF NC-mimic and Gly-tRF mimic in HEK-293T cells. Renilla luciferase activity (R value) was performed to correct and standardize firefly fluorescence activity (F value). Statistical graph data is expressed by F/R of three independent experiments. **F** Western blotting performed to detect the level of NDFIP2 protein in HCC cells stably transfected with Gly-tRF NC-mimic and Gly-tRF mimic. *P < 0.05, **P < 0.01, ****P < 0.0001

Then, we evaluated the effect of Gly-tRF mimic transduction on the expression of selected predicted target genes by qRT-PCR. Nedd4 family interacting protein 2(NDFIP2) is an activator of Nedd4 family E3 ubiquitin ligases [36], and its mRNA level decreased after Gly-tRF mimic transduction (Fig. 4A). Interestingly, the NDFIP2 level was found to be significantly reduced in 371 HCC tissue, compared with 50 normal liver tissues by matching the expression levels of NDFIP2 in the TCGA database (https://portal.gdc.cancer.gov/), and low NDFIP2 expression was found to imply a poor prognosis of HCC based on the Kaplan–Meier Plotter database (http://kmplot.com/) (Fig. 4B, C). Immunohistochemistry was used to verify the expression of NDFIP2 in HCC, and the results showed that 58/84 (69%) of the samples had lower NDFIP2 expression in tumour tissues than in non-tumor tissues (Fig. 4D). These evidences support our preliminary prediction that Gly-tRF may target to regulate NDFIP2 in HCC.

Dual luciferase reporter assays were employed to determine whether Gly-tRF binds to the seed sequence in the NDFIP2 3′ UTR. There were potential targeted binding sites of Gly-tRF on NDFIP2 3′ UTR based on the miRDB database (Fig. 4E). Our results showed that luciferase activity was significantly reduced when HEK-293T cells were co-transduced with Gly-tRF mimic and NDFIP2 wt reporter plasmid (Fig. 4F). However, pGL6 empty vector and NDFIP2 mut reporter plasmid did not inhibit luciferase activity (Fig. 4F).

**Fig. 5:**
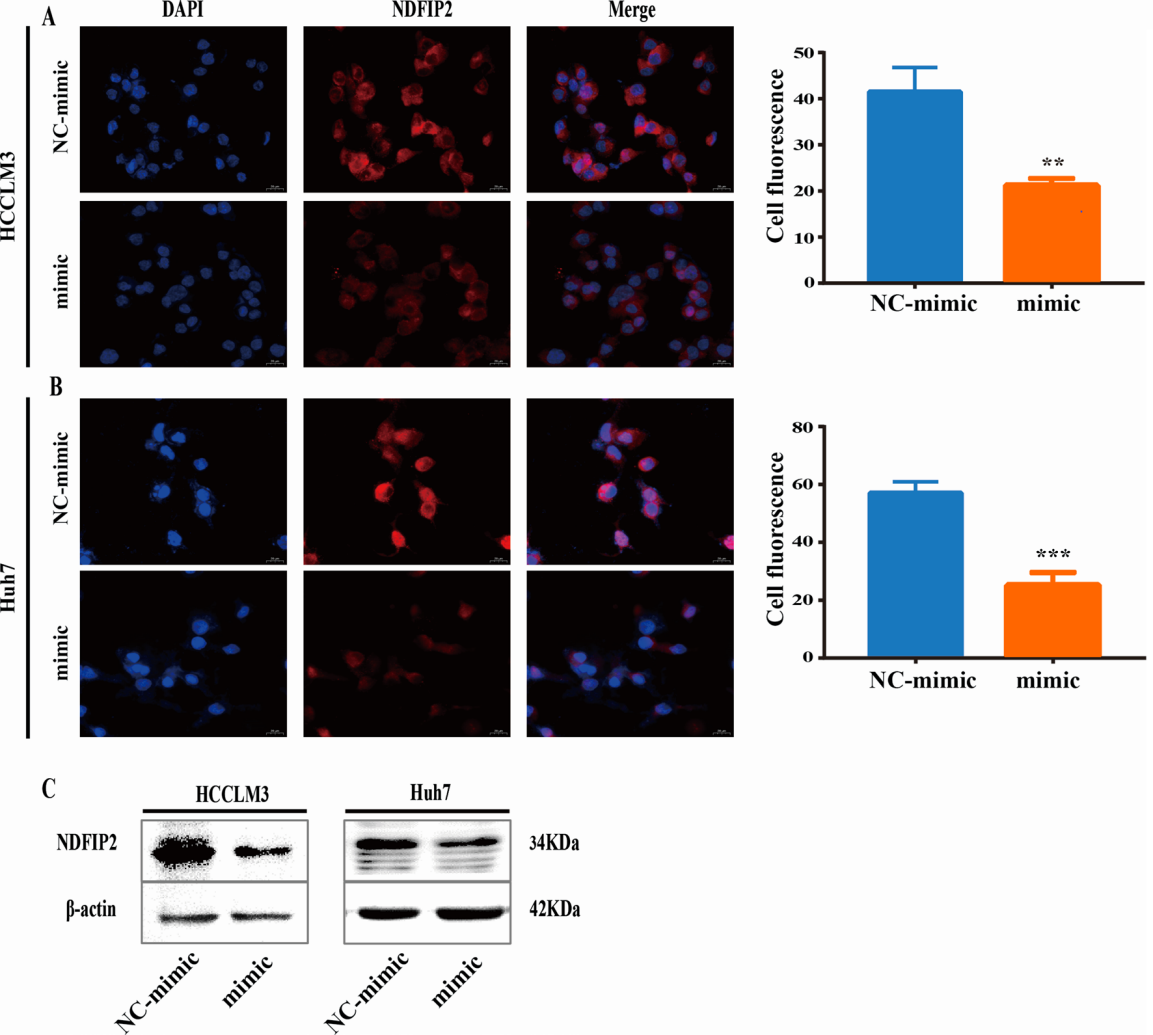
Gly-tRF inhibits the expression level of NDFIP2 protein. **A**–**C** Immunofluorescence and Western blotting were performed to detect the level of NDFIP2 protein in HCC cells stably transfected with Gly-tRF NC-mimic and Gly-tRF mimic. The statistical graph shows the average value of cell fluorescence in 3 random fields. Magnification, 400 × . Data are shown as mean ± SD. **P < 0.01, ***P < 0.001

We detected the effect of Gly-tRF mimic transduction on the protein expression level of NDFIP2 by immunofluorescence and Western blotting analyses. When cells were transduced with the Gly-tRF mimic, the protein expression level of NDFIP2 was decreased (Fig. 5A–C). Our experiments demonstrate that Gly-tRF specifically targets NDFIP2 in HCC.

### Overexpression of NDFIP2 partially reverses the HCC-promoting effect of Gly-tRF

**Fig. 6:**
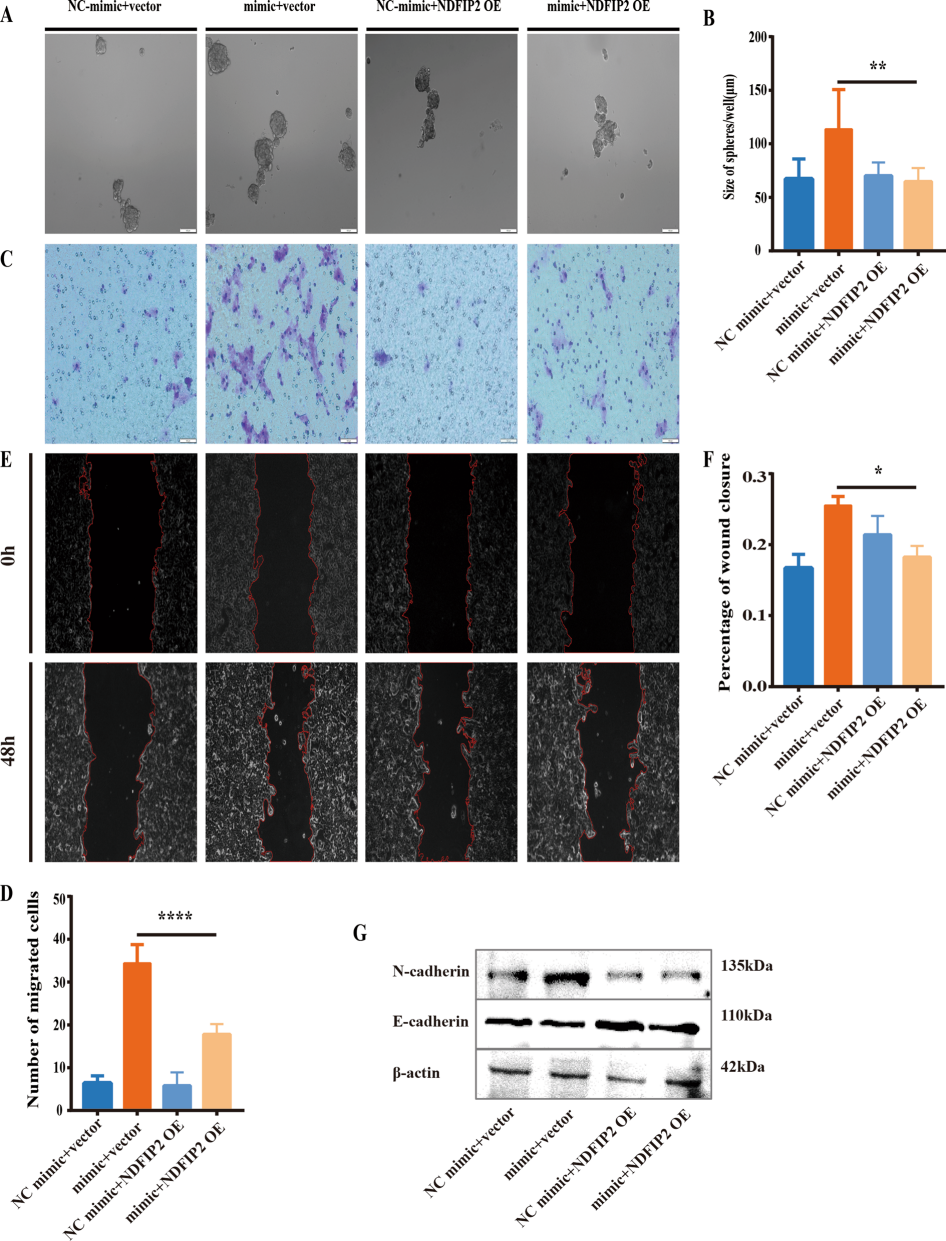
Overexpression of NDFIP2 partially reverses the HCC-promoting effect of Gly-tRF. **A**, **B** The sphere formation assay was performed to verify that the overexpression of NDFIP2 reversed CSCs sphere formation ability caused by Gly-tRF. Magnification, 100 × . **C**-D. The Transwell assay was performed to verify that the overexpression of NDFIP2 reversed the promotion of Gly-tRF on HCC cell migration. Magnification, 200 × . **E**, **F** Wound healing assay was performed to verify that the overexpression of NDFIP2 reversed the promotion of Gly-tRF on HCC cell migration. Magnification, 100 × . **G** Western blotting was performed to detect the abundance of core markers (N-cadherin, E-cadherin) in EMT. Data are shown as mean ± SD. *P < 0.05, **P < 0.01, ****P < 0.0001

Next, we further investigated whether overexpression of NDFIP2 abolishes or reverses the promotive effect of Gly-tRF on the migration of HCC cells and LCSC-like properties. We constructed the NDFIP2 overexpression plasmid, and when the NDFIP2 overexpression plasmid was transfected into HCCLM3 cells, the expression levels of NDFIP2 mRNA and protein were verified to be increased through qRT-PCR and Western blotting analyses (Additional file 2: Fig. S2A, B). Our results showed that overexpression of NDFIP2 reliably reversed the increase in LCSC sphere formation ability induced by Gly-tRF in HCCLM3 cells (Fig. 6A, B). Co-transfection of the NDFIP2 overexpression plasmid with the Gly-tRF mimic abolished Gly-tRF-induced cell migration and EMT-related marker abundance in HCCLM3 cells (Fig. 6C–G). Collectively, these functional reversal experiments further confirmed that Gly-tRF acts as a cancer-promotor by targeting NDFIP2.

### Gly-tRF/NDFIP2 functions by activating the AKT signalling pathway

**Fig. 7:**
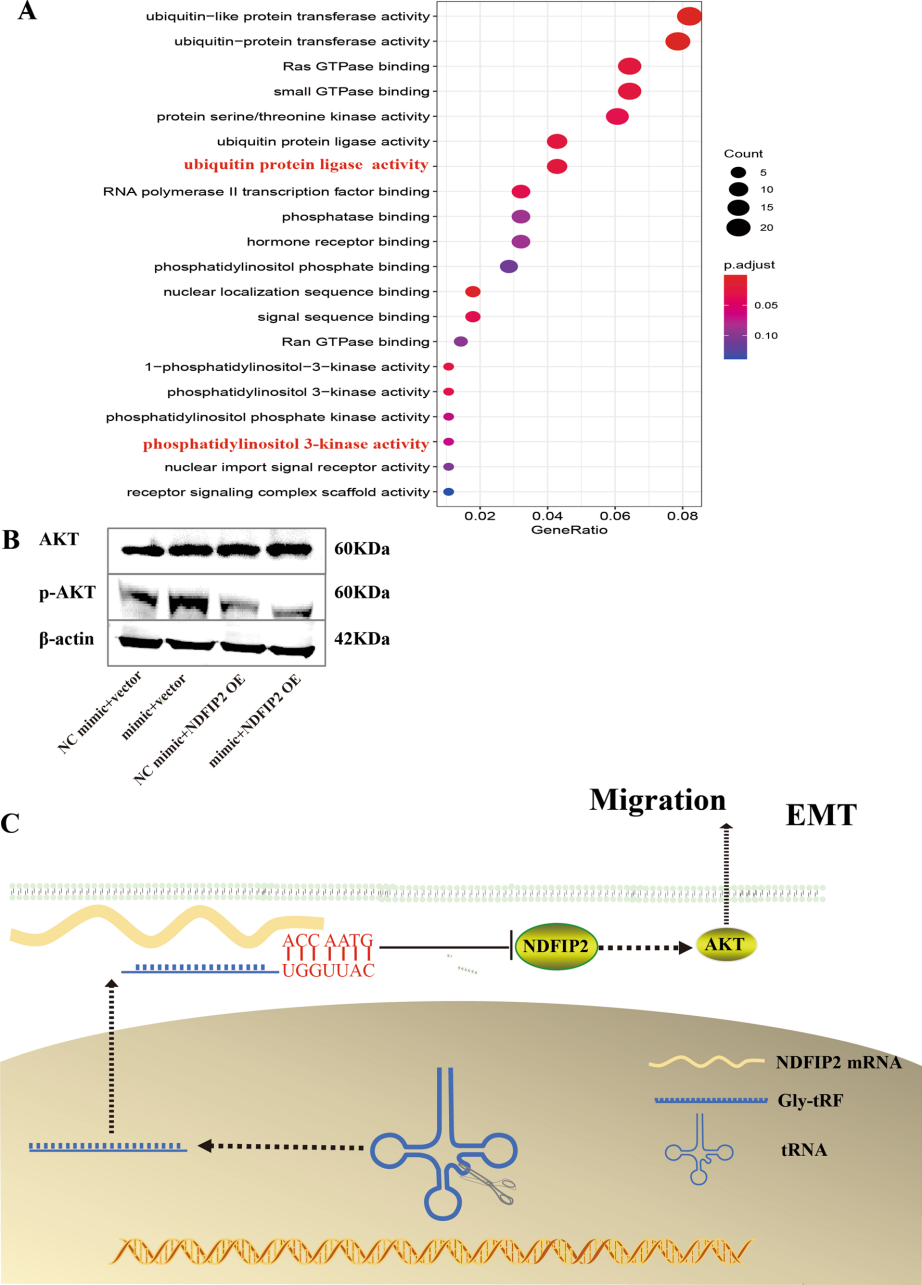
Gly-tRF/NDFIP2 functions through activating the AKT signalling pathway. **A** GO molecular function (GO-MF) analysis of NDFIP2 gene. **B** Western blotting was performed to detect the phosphorylated levels of AKT. **C** Model diagram of Gly-tRF/NDFIP2 axis functioning in HCC

Next, we aimed to identify the signalling pathways through which Gly-tRF/NDFIP2 functions. Through GO molecular function (GO-MF) analysis, we found that Gly-tRF/NDFIP2 is mainly involved in ubiquitin protein ligase activity and phosphatidylinositol 3-kinase (PI3K) activity (Fig. 7A).

Previous studies have shown that depletion of NDFIP2 can inhibit AKT activation, and promote HeLa cell proliferation [37]. Western blotting was used to investigate whether the AKT signalling pathway is involved in the function of NDFIP2. We found that in Gly-tRF mimic transduced HCCLM3 cells, the abundance of phosphorylated AKT was increased and was reversed by overexpression of NDFIP2 (Fig. 7B).

In summary, the above data indicate that Gly-tRF negatively regulates the expression of NDFIP2, thereby activating the AKT signalling pathway to promote HCC (Fig. 7C).

## Discussion

The idea that transfer RNA derived fragments (tRFs and tiRNAs) play key roles in mechanisms of tumorigenesis and tumour development is supported by accumulating research evidence. However, the effect of tRFs and tiRNAs on HCC is still unknown, and further research is needed. In this regard, current research has identified a tRNA derived fragment, Gly-tRF, that is upregulated in HCC cell lines and HCC tissues. Furthermore, our results confirm that elevated Gly-tRF promotes HCC cell migration These results support the important roles of Gly-tRF in the mechanism of HCC, which is of great significance for the development of new research focuses on strategies for the diagnosis and treatment of HCC.

Previous studies have shown the biological function and potential molecular mechanism of dysregulated functional tRFs and tiRNAs in HCC. Deep-sequencing analysis of small RNAs identified a tRF named tRF_U3_1, that exhibits increased abundance in the Huh7 cell line and negatively regulates viral gene expression [38]. High-throughput sequencing results of small RNAs in liver tissues of patients with advanced hepatitis B or C and HCC showed that tRFs and tiRNAs have the highest abundance in chronically infected liver tissue, and that their abundance is changed in HCC [39]. Accumulating evidence reveals the potential relationship between tRFs and HCC. Although the tRNA-derived fragment subtypes and tumour types were different from those studied previously, those of previous studies indicate that elevated levels of tRNA-derived fragments act as a cancer-promoting factor [40–42]. However, the expression levels of tRFs are affected by the cellular context, and the transcription characteristics of tRFs are related to personal attributes [43]. These variations make discovering the function of tRFs extremely complicated. Currently, tRFs represent an emerging, elusive, challenging and promising field, and their regulation of biological activities requires more in-depth evaluation and more convincing evidence.

tRF usually targets mRNA 3′ UTR through miRNA-like effects and plays potential roles in post-transcriptional regulation [44]. The tRF/miR-1280 derived from tRNA^Leu^ and pre-miRNA inhibits Notch signaling pathway by directly targeting Notch ligand JAG2 mRNA 3′ UTR, inhibiting the growth and metastasis of colorectal cancer cells [18]. tRF-17-79MP9PP targets THBS1 3′ UTR to attenuate breast cancer cell invasion and migration [45]. In this study, we confirmed that NDFIP2 is the direct target of Gly-tRF. We also found that the promotive effects of Gly-tRF on HCC can be reduced by NDFIP2 overexpression.

There have been discussions about the role of tRNA-derived fragments in stemness regulation. In mouse stem cell models, 5′ tRNA accumulation has been found to regulate the undifferentiated stem cell status in tumours through differential translation of proteins that regulate cell migration, adhesion, and stress response [46]. PUS7-mediated pseudouridylation activates tRF biogenesis to control protein synthesis and stem cell fate determination, and this post-transcriptional regulatory network directly affects tumorigenesis [47]. The results of the present study indicate that Gly-tRF increases the expression of markers indicating a stem cell-like phenotype and promotes LCSC-like properties. Collectively, the above findings and our results suggest that it is meaningful to integrate the study of tRNA-derived fragments into research on cancer stem cells.

The potential mechanism by which tRNA-derived fragments control the biological processes of HCC cells is multi-step and complex. Importantly, we found that the tumour -promoting effect of Gly-tRF on HCC cells depends on the AKT signalling pathway. Overexpression of NDFIP2 weakened the tumour-promoting effect of Gly-tRF on HCC cells and restored the level of phosphorylated AKT. Accumulating evidence also indicates the activation of the AKT signalling pathway in HCC biogenesis [48, 49]. NDFIP2 regulates the stability of its target proteins by activating E3 ubiquitin ligases [36]. Our GO-MF analysis result also implied that NDFIP2 is involved in ubiquitin mediated proteolysis. We thus speculate that NDFIP2 regulates the AKT signalling pathway through the ubiquitination of downstream target proteins. However, our current research has not verified this speculation. Additionally, the number of human tRFs identified to date exceeds the number of human protein-coding genes, the mechanism of tRFs involved in biogenesis has not yet been elucidated, and multiple mechanisms may be responsible [43].

## Conclusion

Gly-tRF regulates the migration of HCC cells and LCSC-like properties through negative regulation of NDFIP2 and activation of the AKT signalling pathway. This result may provide a theoretical basis for Gly-tRF as a target in future treatment of HCC. However, the small number of clinical samples and the limitations of detection methods may affect the true expression of Gly-tRF. The expression abundance of tRF is affected by the cell environment, and the transcription characteristics are also related to personal attributes, the dynamic changes of tRF involved in disease progression need further study.

## Supplementary Information

**Additional file 1: Figure S1.** A flowchart of the article to show the research methodology.

**Additional file 2: Figure S2.** A-B. Real time PCR (A) and western blotting (B) were used to detect the level of NDFIP2 mRNA and NDFIP2 protein when Gly-tRF NC-mimic and Gly-tRF mimic co-transfected with NDFIP2 overexpression plasmid in HCCLM3 cells. Data are shown as mean ± SD. **P < 0.01, ****P < 0.0001.

## Data Availability

All data supporting this research are included in the article and supplementary materials.

## Abbreviations

tRNA: Transfer RNA
Gly-tRF: Glycine tRNA-derived fragments
HCC: Hepatocellular carcinoma
CSCs: Cancer stem cells
LCSCs: Liver cancer stem cells
NDFIP2: Nedd4 family interacting protein 2
EpCAM: Epithelial cell adhesion molecule
tRFs: TRNA-derived small RNA fragments
tiRNAs: TRNA-derived stress-induced RNA
ncRNAs: Non-coding RNAs
AFLD: Alcoholic fatty liver disease
CCTCC: China Center for Type Culture Collection
STR: Short tandem repeats
DMEM: Dulbecco’s modified Eagle’s medium
FBS: Fetal bovine serum
PBS: Phosphate belanced solution
qRT-PCR: Quantitative real-time polymerase chain reaction
PE: Phycoerythrin
APC: Allophycocyanin
Opti-MEM: Optimized minimum essential medium
DAPI: 4′,6-Diamidino-2-phenylindole
TCGA: The Cancer Genome Atlas
DEGs: Differentially expressed genes
GO: Gene Ontology
ANOVA: Analysis of Variance
NC: Negative control
3′ UTR: 3′-Untranslated regions
GO-MF: GO molecular function
AKT: Protein Kinase B
JNK: C-Jun N-terminal kinase
PUS7: Pseudouridine synthase 7
