# Supplementary figures and images for: Gly-tRF enhances LCSC-like properties and promotes HCC cells migration by targeting NDFIP2
Source: Cancer Cell Int. 2021 Sep 18;21:502. doi: 10.1186/s12935-021-02102-8 (PMC8449465; doi:10.1186/s12935-021-02102-8)

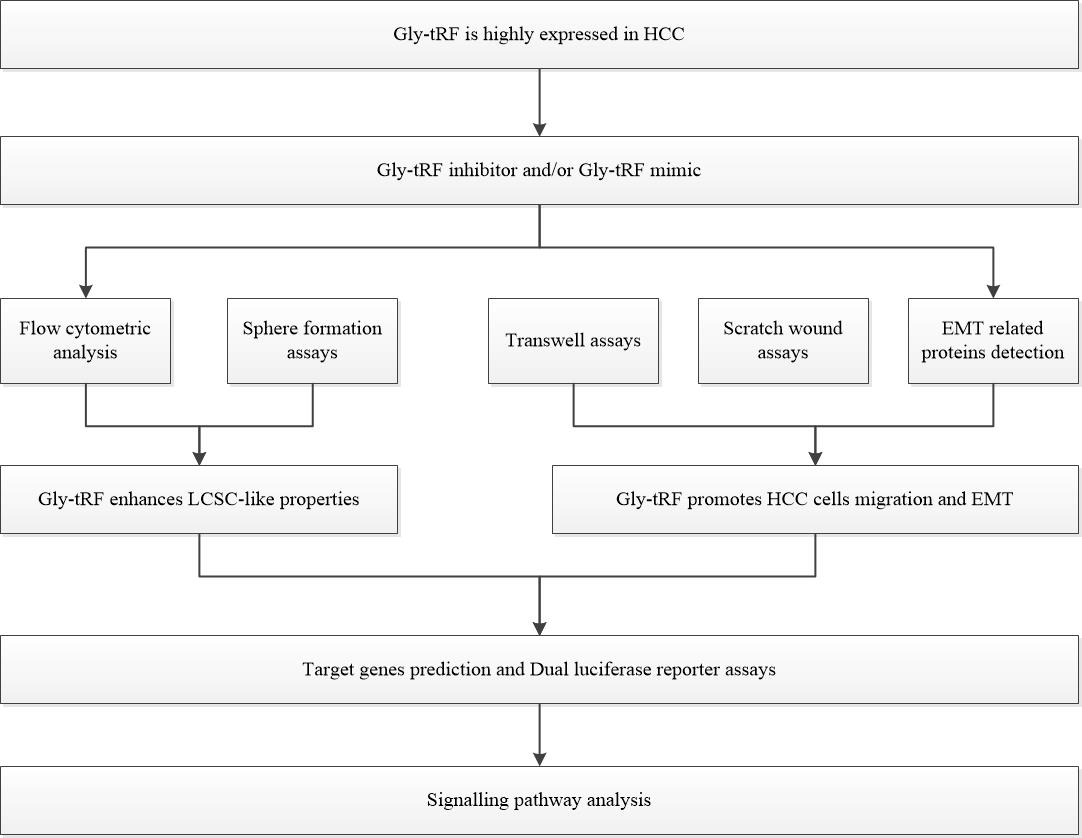

Supplement: Supplementary file 1 — Additional file 1: Figure S1. A flowchart of the article to show the research methodology. [file 12935_2021_2102_MOESM1_ESM.jpg]

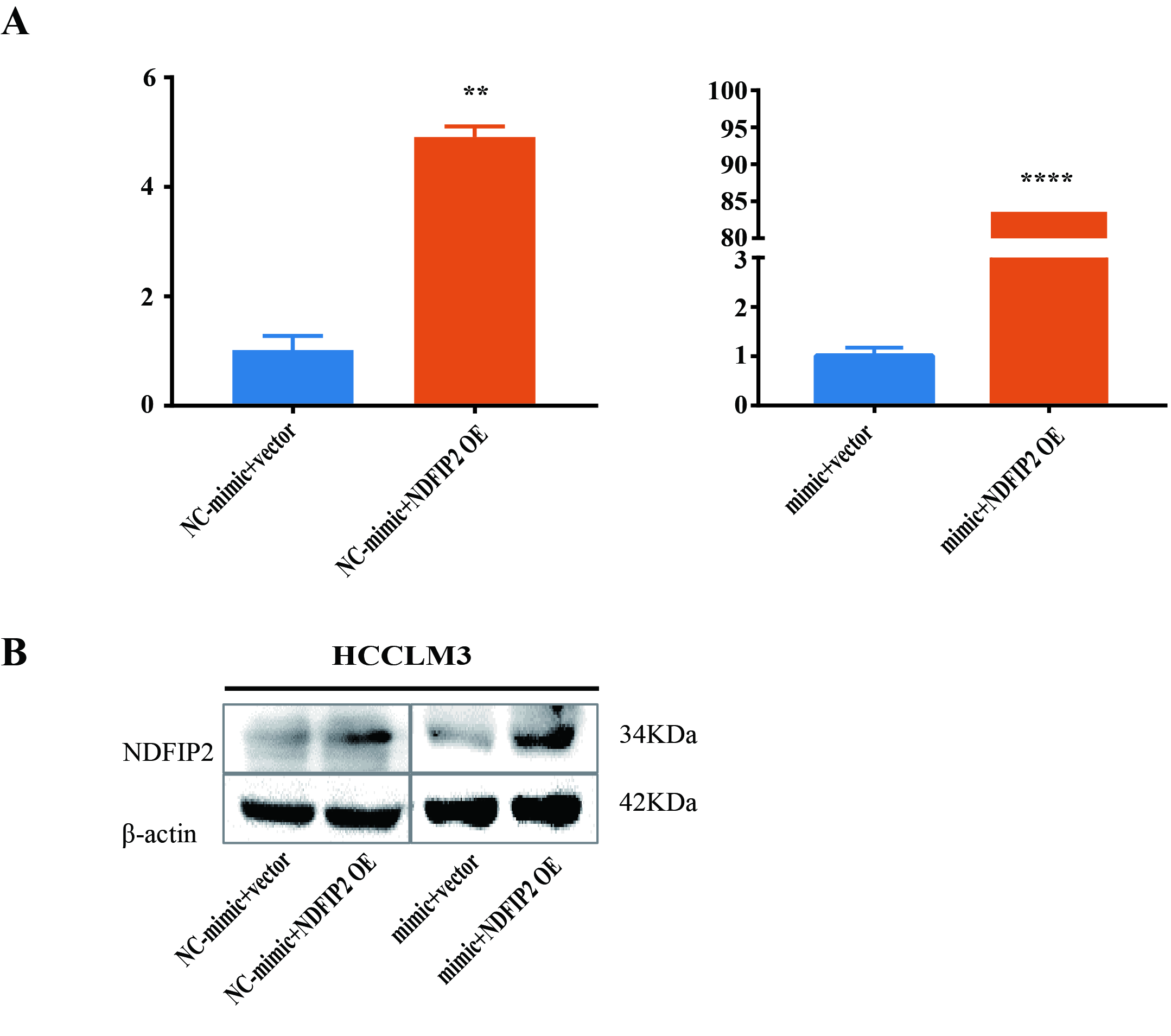

Supplement: Supplementary file 2 — Additional file 2: Figure S2. A-B. Real time PCR (A) and western blotting (B) were used to detect the level of NDFIP2 mRNA and NDFIP2 protein when Gly-tRF NC-mimic and Gly-tRF mimic co-transfected with NDFIP2 overexpression plasmid in HCCLM3 cells. Data are shown as mean ± SD. **P < 0.01, ****P < 0.0001. [file 12935_2021_2102_MOESM2_ESM.tif]
